# SPADs and SiPMs Arrays for Long-Range High-Speed Light Detection and Ranging (LiDAR)

**DOI:** 10.3390/s21113839

**Published:** 2021-06-01

**Authors:** Federica Villa, Fabio Severini, Francesca Madonini, Franco Zappa

**Affiliations:** Dipartimento di Elettronica, Informazione e Bioingegneria—Politecnico di Milano, Piazza Leonardo da Vinci 32, I-20133 Milano, Italy; fabio.severini@polimi.it (F.S.); francesca.madonini@polimi.it (F.M.); franco.zappa@polimi.it (F.Z.)

**Keywords:** light detection and ranging (LiDAR), single photon avalanche diode (SPAD), silicon photo multiplier (SiPM), SPAD array, time-of-flight (TOF), 3D ranging, scanning

## Abstract

Light Detection and Ranging (LiDAR) is a 3D imaging technique, widely used in many applications such as augmented reality, automotive, machine vision, spacecraft navigation and landing. Achieving long-ranges and high-speed, most of all in outdoor applications with strong solar background illumination, are challenging requirements. In the introduction we review different 3D-ranging techniques (stereo-vision, projection with structured light, pulsed-LiDAR, amplitude-modulated continuous-wave LiDAR, frequency-modulated continuous-wave interferometry), illumination schemes (single point and blade scanning, flash-LiDAR) and time-resolved detectors for LiDAR (EM-CCD, I-CCD, APD, SPAD, SiPM). Then, we provide an extensive review of silicon- single photon avalanche diode (SPAD)-based LiDAR detectors (both commercial products and research prototypes) analyzing how each architecture faces the main challenges of LiDAR (i.e., long ranges, centimeter resolution, large field-of-view and high angular resolution, high operation speed, background immunity, eye-safety and multi-camera operation). Recent progresses in 3D stacking technologies provided an important step forward in SPAD array development, allowing to reach smaller pitch, higher pixel count and more complex processing electronics. In the conclusions, we provide some guidelines for the design of next generation SPAD-LiDAR detectors.

## 1. Introduction

Light detection and ranging (LiDAR) is a widespread technique used to reconstruct three-dimensional (3D) scenes for many applications such as augmented and virtual reality [1], automotive [2], industrial machine vision [3], Earth mapping [4], planetary science [5], and spacecraft navigation and landing [6]. Augmented reality is a leading branch, since it exploits applications in many fields such as vehicles navigation, military vision, flight training, healthcare and education. Many technology companies are pursuing great efforts in the automotive field for autonomous vehicles (AVs) and advanced driver assistance systems (ADAS), and the total available LiDAR market for ADAS, AV and trucks is expected to triple between 2025 and 2030, reaching up to USD120 B [7].

In this paper, after a review of the most common LiDAR techniques and illuminations schemes, we will describe requirements and technologies for LiDAR detectors, focusing on single photon avalanche diode (SPAD) and silicon photomultiplier (SiPM) imagers. We will compare different detector architectures by analyzing how they face the most critical LiDAR challenges, to provide guidelines for the design of optimized SPADs and SiPMs imagers for LiDAR.

### 1.1. LiDAR Techniques

LiDAR techniques aim to reconstruct 3D maps of the environments under observation. Different methods for 3D ranging have been proposed, including stereo-vision, projection with structured light and time-of-flight (TOF), the latter being among the most promising one for long-range and real-time applications, and is commonly called LiDAR.

**Figure 1:**
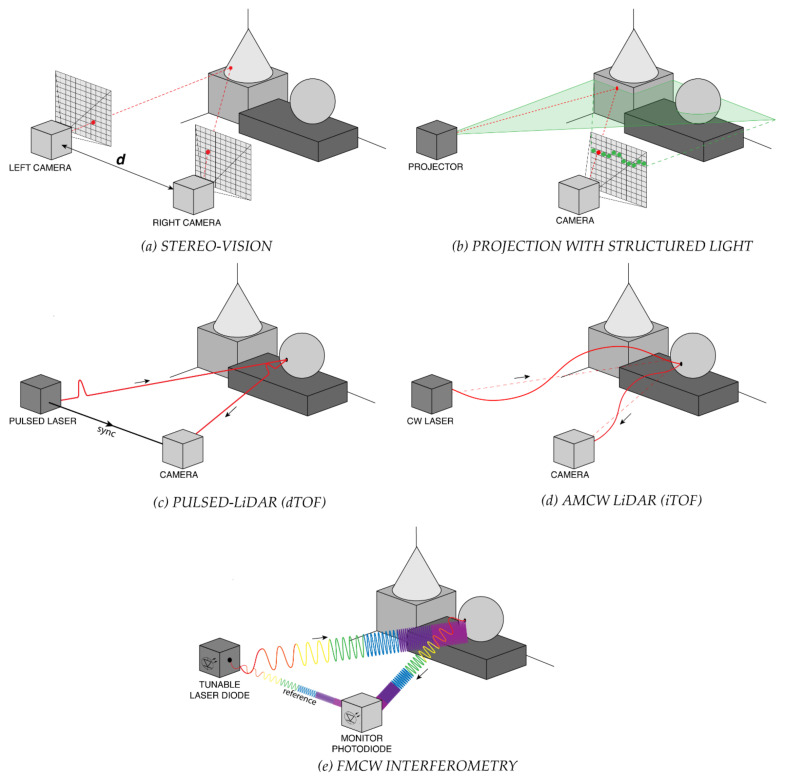
Schematic representation of some 3D ranging techniques: (**a**) stereo-vision, (**b**) projection with structured light, (**c**) pulsed-LiDAR (dTOF), (**d**) amplitude-modulated continuous-wave (AMCW) LiDAR (iTOF), and (**e**) frequency-modulated continuous-wave (FMCW) interferometry.

*Triangulation* exploits trigonometry laws to extract 3D spatial information [8]. In particular stereo vision [9] estimates distances through the simultaneous acquisition of two two-dimensional (2D) images of the same scene, by means of two cameras separated by a known distance *d* (Figure 1a). A sophisticated algorithm (named stereo disparity algorithm [10]) processes the images and computes the distance of the target. It is the same technique used by animals and humans to obtain information on distance and depth of objects in the surrounding environment. The advantage of stereo-vision is that it achieves high resolution and simultaneous acquisition of the entire range image in a simple passive-way, i.e., with neither active illumination nor energy emission. However, this method requires solving the so-called correspondence problem (identification of point pairs, which are projections of the same point in the scene), which is computationally expensive and results in a limited frame-rate [11]. Furthermore, stereo-vision is a non-robust technique, because of the parallax method used to measure distances. In fact, it fails if a nearby object covers a distant one in one of the two images; in that case, it is not possible to measure the further distance and the 3D image has a loss of information. The maximum Full-Scale-Range (FSR) depends on the baseline between the two cameras and the larger the base line the longer the range, but also the further the sensing range. Commercially available stereo vision cameras have typical operating distances of few meters (3 m to 5 m) [12,13,14].

Unlike stereo vision, projection with *structured light* requires an active illumination source, which shines a predetermined pattern of light (usually horizontal or vertical lines or spots, as shown in Figure 1b) toward the objects in the scene [15]. The 2D image is acquired and, analyzing how the light pattern gets modified by the targets, it is possible to reconstruct their 3D distance and shape. This technique is very accurate and precise (it can achieve sub-millimeter resolution), but it is slow because of the algorithm complexity, hence it cannot provide real-time acquisitions. Moreover, to improve depth resolution the pattern is changed several times (up to ten for every scene) and for each one the acquisition and the processing are repeated. The camera can also be moved around the scene, so to perform a high number of acquisitions, but lowering the overall measurement speed. For these reasons, structured light is used in applications where precision is a fundamental requirement instead of speed. Furthermore, the achievable FSR of commercially available structured light cameras is limited to few meters [16,17,18], which is another strong limitation in many applications. An example of commercial device based on structured light is the well-known Kinect v1 (Microsoft, Redmond, WA, USA) which can acquire 3D images with 5 mm precision up to 3.5 m range [19].

TOF techniques measure the path travelled by an excitation light through a medium to reach the target object and to return to the detector. They are widely exploited not only in LiDAR, but also in bioimaging [20]. Differently from stereo vision and structured-light projection, TOF-LiDAR does not require complex reconstruction algorithms, thus it enables real-time applications. Moreover, TOF techniques are the most suitable ones when large field of view (FOV) and centimeter resolution are needed. TOF can be directly measured, with time-resolved detectors and electronics (pulsed-LiDAR) or indirectly estimated through phase-resolved measurements (continuous wave or CW-LiDAR). 

*Pulsed-LiDAR*, also known as *direct-TOF* (dTOF), relies on the measurement of the round-trip travel time of an optical pulse hitting and being back-scattered from a distant target (Figure 1c). From the TOF timing information, the distance D of the target is computed through the light velocity c in the medium, namely D = ½ · c · TOF. This technique requires short (usually <1 ns) laser pulses, high-bandwidth detectors and timing electronics with sub-nanosecond resolution and time jitter. In pulsed-LiDAR, the resolution of the electronics (σ_TOF_) directly affects the depth resolution, being σ_d_ = ½ · c · σ_TOF_. The distance FSR is only limited by the illuminator power, the target reflectivity and the timing electronics FSR. For this reason, pulsed-LiDAR is particularly suitable in applications requiring long ranges (up to kilometers). 

In order to acquire long-range distances by employing low energy laser pulses and low reflectivity targets, single photon detectors together with photon-timing (e.g., a time-to-digital converter, TDC) and photon-counting (e.g., a digital gated counter) electronics become a must. In those cases, the laser return will no longer be an analog signal (e.g., photocurrent or photoelectron charge packet), but a digital pulse (e.g., a rising-edge logic transition when at least one photon gets detected). In the following we will mainly focus on these devices. In case of repetitive TOF measurements at the single-photon level, such technique is also known as time-correlated single-photon counting (TCSPC), able to reconstruct the shape of very faint and fast (down to the ps range) light signal [21], such as in fluorescence [22] and spectroscopy [23]. 

**Figure 2:**
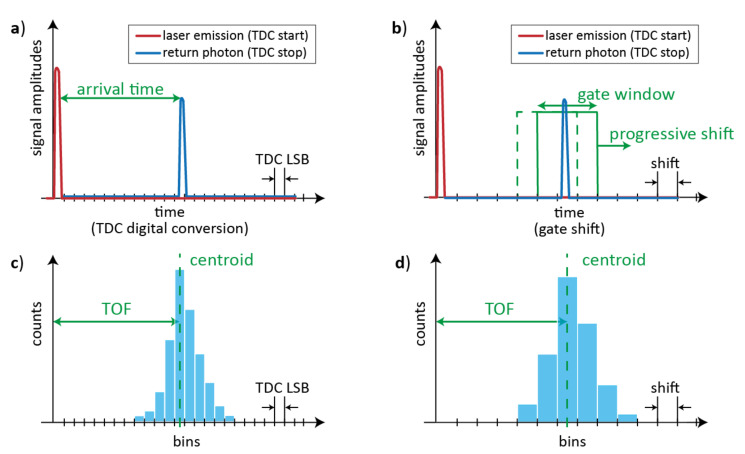
Schematic representation of different pulsed-LiDAR approaches: (**a**) a TDC measures the arrival-time of incoming photons (the time axis is quantized by the TDC’s Least Significant Bit, LSB); (**b**) a gated-detector measures the signal intensity within a gate window that is progressively-scanned across the range (the time axis is quantized by the gate shifts). In both cases, the histogram of collected data, either TDC timing information (**c**) or measured intensities (**d**), is used to extract the distribution centroid, corresponding to the average TOF.

As shown in Figure 2, pulsed-LiDAR systems typically employ either TDCs to timestamp the arrival-time of the laser pulse echo (Figure 2a) or gated-detectors to measure the signal intensity within short gate windows, progressively spanning across the FSR with increasing gate delays from the laser pulse emission (Figure 2b). Both techniques can be applied also with very dim signals at single photon rate, repeating many times the measurements in order to build a histogram of the acquired data (either TOFs or intensities), whose bin-width depends on either TDC resolution or gate window delay shift, respectively (Figure 2c,d). Each measurement can look at a single spot of the scene or at the full scene, depending on the optics and if a single-pixel detector or a multi-pixel imager is employed; anyhow, usually a histogram is accumulated per each pixel. The computation of the histogram centroid gives the average TOF, hence the distance of the spatial spot imaged by the LiDAR’s optics. Thus information about the target distance and shape can also be extracted from the histogram of the reflected signal.

The TDC approach is sensitive to all photons returning within the FSR, whereas the gated-detector approach counts only photons returning within the selected gate window, with the advantage of time filtering the incoming light and reduce the distortion due to background illumination. On the other hand, time-gating drastically reduces the actual detection efficiency of the measurement, therefore, progressive-scanning requires long measurement times regardless of the background conditions, and it results hardly compatible with real-time applications and long ranges. Vice versa, the TDC approach could be limited by either the maximum number of TDC conversions and storage availability per laser pulse or the dead-time of both TDC and single-photon detector (i.e., the time required to be ready for a next conversion or detection). Therefore, compared to the ideal case of 100% detection efficiency, no dead-times, multi-hit TDC electronics, actual pulsed-LiDAR systems may have major performance limitations.

In *continuous-wave CW-LiDAR*, instead of emitting short high-energy laser pulses, either amplitude modulated (AMCW) or frequency modulated (FMCW) light is employed. AMCW-LiDAR is achieved through the so-called indirect-TOF (iTOF) technique, which relies on measurement of the phase difference between an amplitude modulated light source and the detected backscattered echo signal (Figure 1d). The excitation signal can be either a sinusoidally amplitude modulated light or a light pulse (with hundreds of nanoseconds pulse width) from lasers or LEDs. In the sinusoidal modulation approach, the echo signal is phase-shifted with respect to the emitted one, by a quantity proportional to the source modulation frequency f and the object distance. From the phase shift ΔФ, it is then possible to infer the distance D = c ∙ΔΦ/4πf. Usually ΔФ is measured by sampling the sinusoidal echo intensity in four equally spaced points (C_0_, C_1_, C_2_ and C_3_) and by computing $\Delta \phi =\mathrm{arctg}\left(\frac{C3-C1}{C0-C2}\right)$ [24]. 

In the pulsed modulation approach, a laser source emits light pulses with duration T_p_ of few hundreds of nanoseconds (usually proportional to the desired FSR) and the back-scattered light is integrated within three time windows, with same width but delayed in time. The first one integrates the whole signal (background plus echo pulse), the second one integrates the same background but only a portion of the echo pulse; the third time window integrates just the background light, which is then subtracted from the two previous measurements. The ratio between the two resulting intensities (multiplied by 2π) provides the phase shift ΔФ. In this way, the measurement is independent of background light, illumination power and object reflectivity, while in AMCW-LiDAR the resolution strongly depends on all of them. Eventually, the distance is computed as D=½·T_p_·$\left(1-\frac{\Delta \phi }{2\pi }\right)$ [25]. Usually, the FSR is limited by the modulation period: e.g., 100 ns pulses or 10 MHz modulation allow to reach 100 ns FSR, i.e., 15 m distance. Nevertheless, methods based on multiple modulation frequencies or linearly chirped modulations have been implemented to extend the unambiguous measurement range [26].

In frequency-modulated FMCW-LiDAR, the laser frequency (i.e., the laser wavelength) is modulated. Typically, the modulation consists of a linear chirp with a small portion of the laser beam (reference) used as local oscillator for the heterodyne demodulation of the return echo signal, as shown in Figure 1e. The modulation bandwidth is often wider than the linearly-chirped AMCW-LiDAR one, yielding to improved depth resolution [27]. Detection requires an optical heterodyne measurement to exploit the interference between emitted and backscattered optical waves, for example through a standard or p-i-n photodiode to demodulate the return signal and generate a beat frequency, which can be recorded by “slow” electronics. The achievable precision is typically much better than in pulsed-LiDAR (dTOF), with the major advantage of using low-bandwidth electronics and cost-effective detectors. Another advantage of FMCW-LiDAR is its ability to directly measure the velocity of the target object by extracting the Doppler-shift generated from the motion [28]. The main limitation of FMCW-LiDAR is the demanding long coherence length of the laser, as it influences the stability of the local oscillator in respect to the backscattered wave, introducing phase-noise. If the measured distance is shorter than the laser coherence length, the peak of the beat-frequency spectrum results sharp and narrow, otherwise the peak widens and its amplitude decreases. Thus the laser coherence length limits the achievable distance FSR. However, FMCW-LiDAR systems able to reach up to 300 m distance range have been recently announced by different companies for automotive applications [29,30].

**Figure 3:**
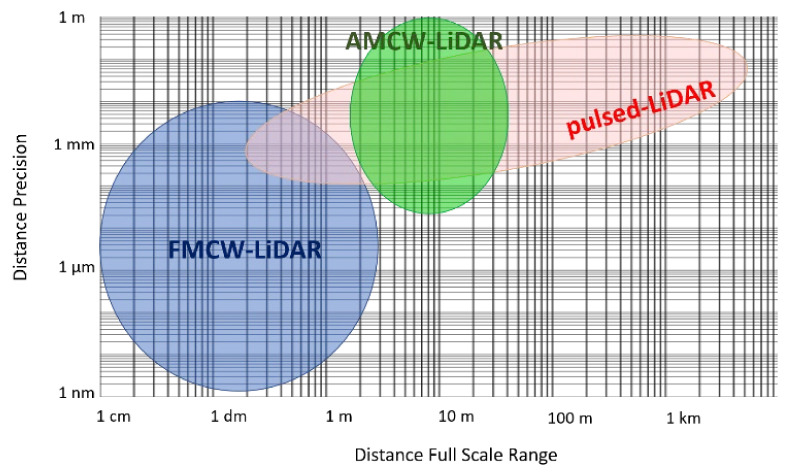
Typical distance Precision vs. Full-Scale Range in pulsed-LiDAR, AMCW-LiDAR and FMCW-LiDAR systems reported so far. Figure adapted from [11].

Only LiDAR techniques can reach long ranges, since both stereo-vision and structured-light approaches are limited to few meters. Figure 3 shows the typical trade-off between precision and FSR of pulsed-LiDAR, AMCW-LiDAR and FMCW-LiDAR systems published starting from 1990 [11]. We can observe that FMCW-LiDAR allows to reach the best precision, but typically at short ranges. Recent commercial FMCW-LiDAR systems which reach long-ranges (up to 300 m) [29,30] have not been reported in Figure 3 since the achieved distance precision is never mentioned. Pulsed-LiDAR is the only one reaching long distances (up to few kilometers) in real-time, a mandatory requirement in many fields such as automotive. For this reason, in this paper we will mainly focus on pulsed-LiDAR (dTOF) systems at the single-photon level.

### 1.2. Illumination Schemes

**Figure 4:**
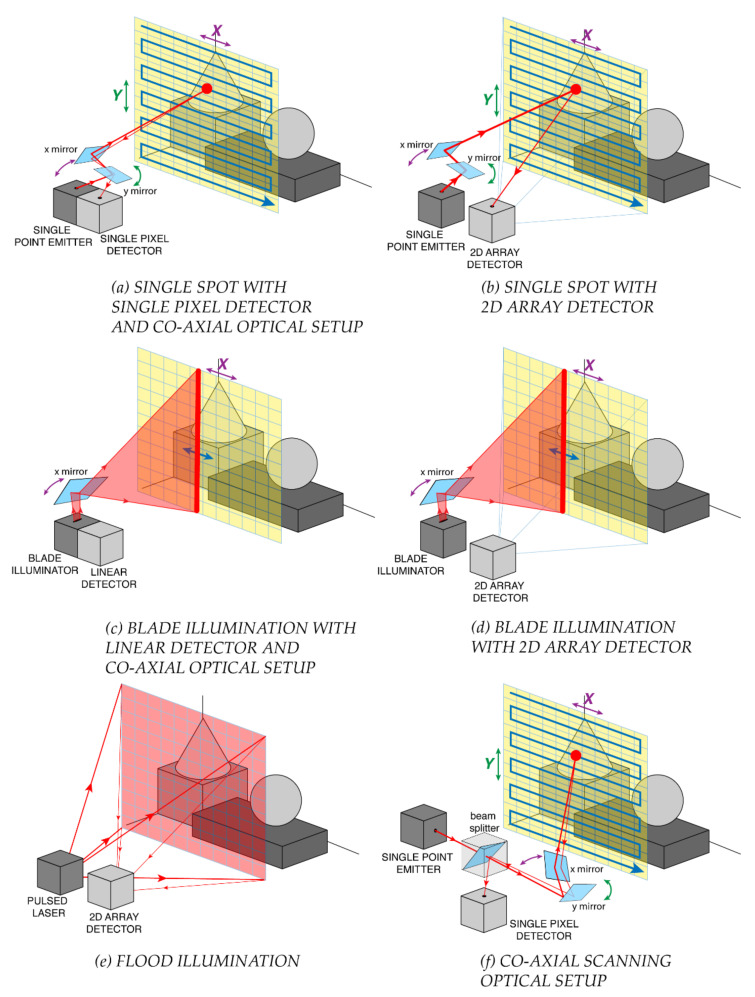
TOF-LiDAR illumination schemes: (**a**) 2D raster scan with single spot and one pixel detector in a co-axial optical setup, (**b**) 2D raster scan with single spot and a 2D array detector, (**c**) 1D line scan with blade beam and a linear array detector in a co-axial optical setup, (**d**) 1D line scan with blade beam and a 2D array detector, (**e**) no scan with flood illumination and 2D imager (full scene flash acquisition), for flash-LiDAR. (**f**) Example of a co-axial scanning optical setup.

TOF-LiDAR techniques require to illuminate the scene by means of either a single light spot, a blade beam, or a flood illumination, as shown in Figure 4. The former two need to cover the whole scene through respectively 2D or 1D scanning, whereas the latter is exploited in *flash-LiDAR* with no need of scanning elements. 

Single-spot illumination typically employs also a single pixel detector (Figure 4a); hence, a coaxial optical system (as shown in Figure 4f) is preferred, in order to avoid any alignment and parallax issue. Note that such a single pixel may be composed by a set (e.g., an array) of detectors, all acting as an ensemble detector (i.e., with no possibility to provide further spatial information within the active area), such as in silicon photomultipliers (see Section 2.1). Alternatively, it is also possible to use a 2D staring array detector (with an active area larger than the laser spot), shining the illumination spot onto the target through a simple non-coaxial optical setup, and eventually measuring the signal echo which walk across the 2D detector, depending also on the object distance (Figure 4b). The main disadvantage is a lower signal-to-noise ratio (SNR) because the detector collects the laser echo from the target spot but also the background light from a larger surrounding area. A more complex detector architecture or readout electronics can recover the ideal SNR by properly tracking the laser spot position within the 2D array detector, so to discard off-spot pixels, for example as proposed in [31].

Blade illumination can be employed in combination with linear detector arrays, mechanically spinning around their axis so to speed up scanning, or using a co-axial optical system (Figure 4c) such as in the Velodyne LiDAR system [32]. Also in this case, it is possible to scan only the blade illumination, while keeping fixed a 2D staring array detector imaging the overall scene, or activating only one row at a time (Figure 4d). 

Finally, flash-LiDAR relies on a flood illumination of the whole scene, while using a staring camera where each pixel images one specific spot of the scene and measures the corresponding distance (Figure 4e). The name “flash” highlights the possibility to acquire images at very high framerates, ideally also in a single laser shot, since no scanning is needed. However, the required laser pulse energy is typically extremely high for covering a sufficiently wide FOV, often far exceeding the eye-safety limits in case of human crossing the FOV at very short distance.

When scanning is required, the beam-steering can be performed with either optomechanical elements (e.g., rotating mirrors and prisms) and electromechanical moving parts (e.g., electric motors with mechanical stages) or compact micro electro-mechanical systems (MEMS) mirrors and solid-state optical phase arrays (OPAs). MEMS and OPAs offer a more compact and lightweight alternative to electromechanical scanning and consequently also faster scanning, for instance through the usage of resonant mirrors. MEMS technology is by far more mature than OPAs, so to become the most exploited technology in modern LiDAR scanning systems [33].

The selection of the illumination scheme must trade off many parameters, such as laser energy, repetition frequency, eye-safety limits, detector architecture, measurement speed, and system complexity. Indeed, compared to scanning techniques, flood illumination in flash-LiDAR typically requires higher laser power to illuminate the entire scene in one shot and assuring enough signal return onto the detector. Such flood illumination could be convenient for eye-safety considerations (if no human being stays too close to the LiDAR output port) because, even if the total emitted power is high, it is spread across a wider area, so the power for unit area could be lower than single-spot long-range illumination. Flash-LiDAR has the advantage of simpler optics, at the expense of large pixel number 2D detector. In fact, the number of pixels limits the angular resolution given the FOV, or vice versa limits the FOV given the angular resolution. Instead, scanning negatively impacts acquisition speed and framerate: particularly 2D scanning is very slow and hardly compatible with real-time acquisitions and fast-moving targets. However, also flash-LiDAR can be operated not single-shot but with more laser shots and image acquisitions to collect enough signal, because the total pulse energy is distributed across a wide FOV and the return signal (above all from far-away objects) can be extremely very faint. In the following, we will focus on both 1D linear scanning with blade illumination and flash-LiDAR with flood illumination, because there is no preferred one for all applications.

### 1.3. Pulsed-LiDAR Requirements and Challenges

The main LiDAR requirements are related to FSR, precision, FOV, angular resolution, and acquisition speed. Furthermore, demanding challenges for outdoor applications are immunity to strong background illuminations, eye-safety, and multi-camera operation (i.e., capability to avoid interference among multiple LiDAR systems).

Regarding the FSR, LiDAR systems can be classified in short range (up to few meters, e.g., in augmented reality for gaming), medium range (up to few tens of meters, e.g., in industrial automation), and long range (hundreds of meters or kilometers, e.g., automotive and satellite vision). Long ranges can be reached only through pulsed-LiDAR techniques in combination with high-power lasers, detectors with single-photon sensitivity, and timing electronics (TDC) with sufficient FSR [34]. 

In pulsed-LiDAR, measurement precision is strongly affected by TDC resolution (i.e., LSB) and minimum feasible delay shift between gate windows: hence, FSR trades-off with precision. Furthermore, precision can be improved by acquiring more arrival-times and computing the centroid of them over many repetitive measurements [35]. 

FOV and angular resolution in scanning systems depend on the scanning speed and number; in flash-LiDAR, they depend on the pixels count. Acquisition speed, a fundamental requirement in real-time applications, is typically related to FOV and angular resolution; in fact, in scanning systems the higher the FOV and the angular resolution, the lower the speed. The same trade-off applies also in flash-LiDAR because, given a certain laser power, larger FOV and higher pixels number result in less photons per pixel, thus the need of repeating more times the acquisition so to get enough signal. In high framerate pulsed-LiDAR, as many photons as possible must be detected and timestamped, thus acquisition speed is also related to overall detection efficiency, maximum count rate, and conversion rate.

Dynamic range (DR) is one of the most challenging detection requirements, above all when operating with strong background light (e.g., outdoor with solar illumination) and with different targets at very different distances and with extremely variable reflectivity and angle. Furthermore, background rejection (i.e., the ability to discard background light from the laser return) is one of the most appreciable features in high-performance LiDAR systems.

**Figure 5:**
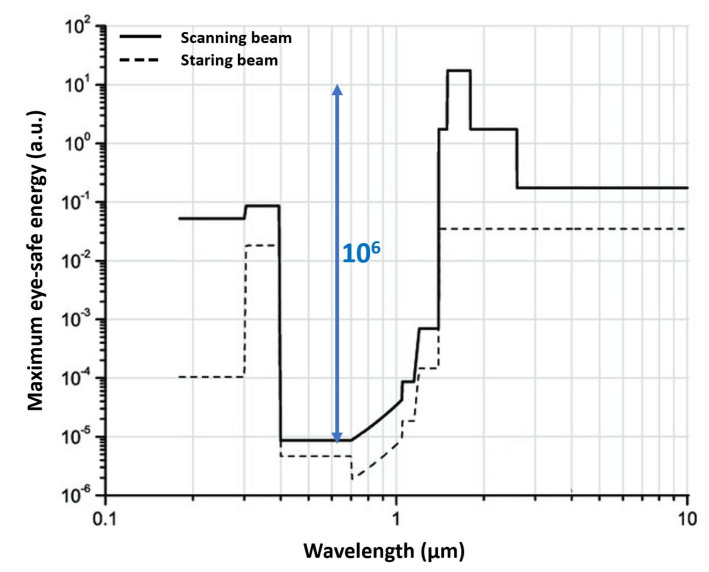
Eye-safe maximum energy vs. wavelength, as required by the American National Standard for Safe Use of Lasers [36]. The maximum energy is given in arbitrary units, since its absolute value depends on beam diameter and pulse width duration.

Concerning regulations, LiDAR systems must be eye-safe, that is the laser must have a safe maximum energy, given the beam diameter, pulse duration and wavelength [36]: for example, Figure 5 clearly shows that working above 1.4 µm wavelength allows to use much higher energies, up to 10^6^ higher than in the visible range. Nevertheless, eye-safety is not the only criterion to select the illumination wavelength; also detector performance and cost, laser availability and cost, and medium (e.g., air, fog, rain, water) transmissivity must be taken into account [37]. High detection efficiency and single-photon sensitivity are desired performance, both for eye-safety considerations and optical power reduction; unfortunately, they usually negatively impact dynamic range and background rejection. 

Finally, immunity to interference among multiple LiDAR devices is another challenge to be faced to deploy reliable LiDAR systems in actual field applications. For instance, in automotive applications LiDAR systems on different vehicles should never interfere when they face each other.

## 2. Detectors for Pulsed-LiDAR Systems

In this section we compare different sensors suitable for pulsed-LiDAR that, according to Section 1.1, is the promising technique for long-range real-time applications. In the comparison, we will take into account all requirements highlighted in Section 1.3.

### 2.1. Detector Technologies

Detectors typically used in pulsed-LiDAR applications include Charge-coupled devices (CCDs), particularly electron-multiplying CCDs (EM-CCDs) [38] and intensified-CCDs (I-CCDs) [39], avalanche photodiodes (APDs) [40], single photon avalanche diodes (SPADs), and silicon photomultipliers (SiPMs), both analog (a-SiPM) and digital (d-SiPM) [41,42,43,44,45,46,47,48,49,50,51,52,53,54,55,56,57,58]. 

SPADs are single photon detectors able to detect at most 1 photon at a time and require a deadtime of some nanoseconds after each photon detection, thus limiting the maximum count rate to about 100 MHz. SiPMs consist in the parallel connection of many microcells, each one composed by a SPAD and its frontend circuit. Therefore, SiPMs are photon number resolved detectors, i.e., they provide information on how many photons got concurrently detected, so they can reach higher count rates than single SPADs, proportionally to the number of microcells (i.e., SPADs): e.g., a SiPM with 100 SPADs can reach a count rate approximately 100 times higher than a single SPAD. In a-SiPMs, the SPAD frontend is just a quenching resistor connected between the SPAD anode or cathode and the common output node shared among all microcells. The a-SiPM output is an analog current given by the sum of the avalanche current pulses from all microcells; hence, the output intensity depends on the number of triggered microcells (i.e., detected photons). In d-SiPM, the SPAD frontend is an active circuit which sets a fixed and well-defined deadtime after each ignition and provides a digital pulse when a photon is detected. The output of all microcells are combined through logic gates (typically an OR-tree). Both a-SiPM and d-SiPM are not spatially-resolved, i.e., they provide an information about the overall signal intensity (detected photons), but no spatial information regarding which microcell got triggered. 

SPAD arrays are detectors with many independent pixels, each one including one SPAD, its active frontend circuitry and typically also a processing electronics (e.g., counters or TDCs) to provide the cumulative number of detected photons or the arrival time of the detected photon. Thus, in SPAD arrays the image resolution corresponds to the number of SPADs. Eventually, SiPM arrays are detectors where each pixel is a SiPM (either a-SiPM or d-SiPM); each pixel can provide spatial information, hence the imager spatial resolution is given by the number of SiPMs and not by the number of microcells. Compared to SPAD arrays, SiPM arrays typically have lower spatial resolution, but each pixel is photon number resolved (being composed by many SPADs). 

**Figure 6:**
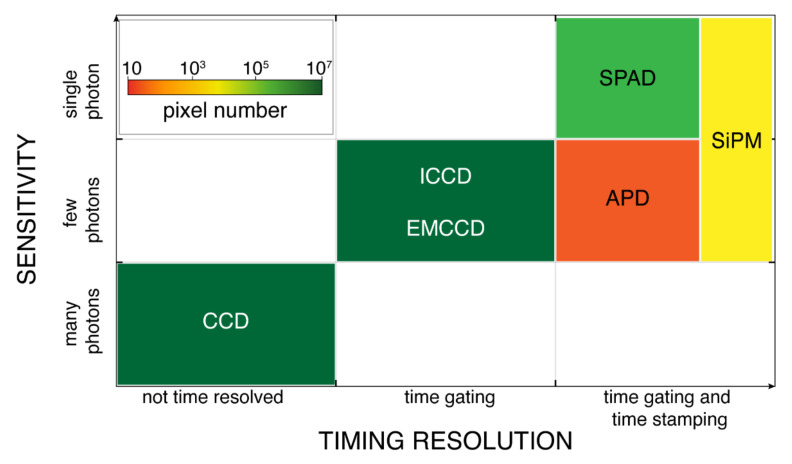
Detectors suitable for LiDAR, compared by timing resolution, sensitivity, and pixel count.

Figure 6 compares the different detector technologies in terms of timing-resolution, sensitivity, and pixels number. Indeed, timing-resolution is a key parameter in measuring photons’ arrival-times in pulsed-LiDAR; sensitivity plays a fundamental role in long range measurements (especially with low reflectivity targets) with limited (eye-safe) laser energy; pixels number impacts FOV, angular resolution, and measurement speed, so to enable flash-LiDAR with no need for scanning elements.

Concerning timing resolution (Figure 6 horizontal axis), we classify detectors in three families: those with no timing capability (photons accumulate over relatively long acquisition times, in the order of tens of microseconds); time-gateable detectors (photons accumulate only during well-defined short time windows, of few nanoseconds); time-stamping detector (coupled with a TDC to directly timestamp photons’ arrival times). Concerning sensitivity (Figure 6 vertical axis), we group detectors sensitive either to many incoming photons (e.g., with minimum detectable signal of hundreds of photons), to few photons (e.g., with minimum detectable signal of few tens of photons), and to a single photon (i.e., the minimum detectable signal is one photon). The available number of pixels of typical detectors is shown in Figure 6 through a color scale: single-pixel detectors are in red; detector arrays with tens or hundreds of pixels are shown in yellow and orange, respectively; kilo-pixels or mega-pixels imagers are in different tonalities of green.

As discussed in Section 1.1, pulsed-LiDAR systems can measure TOF either by time-stamping the incoming photons’ arrival-time through a TDC or by counting photons within gate widows, which are then progressively delayed (shifted in the time domain) with quantized steps across the FSR. The former approach is convenient in most applications, since the detector is active during the entire FSR; whereas the latter is less efficient, since all photons arriving outside the gate window are lost, with the consequence that the measurement time must be increases to attain a sufficient signal and histogram statistics quality. Therefore, detectors with both time-gating and time-stamping capabilities, such as APDs, SPADs and SiPMs are preferred for pulsed-LiDAR; and among them, only SPADs and SiPMs have single photon sensitivity. Furthermore, the design of large APDs arrays is critical, since APDs require dedicated technologies, large pixel pitch, fast analog fronted and high voltage operation [59]. Instead, imagers based on arrays of SPADs or SiPMs composed by very many SPADs in parallel are easy to deploy and have been reported by many research groups and companies. 

Single photon sensitivity is particularly important in long-ranges applications, in fact the photon number reaching the detector pixels depends on the inverse of square distance, as well as on optical parameters (source power and divergence, objective f-number and lenses attenuation), target reflectivity and detector geometry (fill-factor and pixel area), as demonstrated in [60]. Considering for instance 800 mW source power, 200 m target distance, 2° source divergence, 2.8 f-number, 10% lenses attenuation, 90% target reflectivity, 80% detector fill-factor and 20 µm pixel pitch, and applying the equations demonstrated in [60] the resulting pixel photon rate is only 1 photon every 1 µs. 

Recent advances in 3D stacking integration applied to SPADs [61] enabled the development of SPAD imagers with pitch smaller than 10 µm [62,63], down to 2.2 µm [64], and also high number of pixels (hundreds of kilo-pixels up to mega-pixels), without degrading the SPAD performance in terms of efficiency, noise and timing jitter [61,62,63,64]. Each SPAD is connected to an independent sensing (and possibly processing) electronics so to act as a pixel of a SPAD array; alternatively, more SPADs are grouped together to share a common processing electronics (e.g., a TDC) so to form pixel of a SiPM array. In other words, in SPAD arrays each pixel contains only one SPAD (thus achieving the narrower angular resolution in LiDAR applications), whereas in SiPM arrays each pixel (defined as the smallest independent unit providing spatial information) includes multiple SPADs (microcells), thus limiting (widening) angular resolution. Compared to SPAD arrays, SiPM arrays are capable to detect coincident photons within the same pixel (i.e., referring to Figure 6, they provide both single- and also few-photons sensitivity), thus enabling single-shot LiDAR. In many cases, the classification between SPAD arrays and SiPM arrays is not trivial, because intermediate solutions are frequently proposed to exploit the advantages of both SPAD arrays (fine angular resolution) and SiPM arrays (single and multi-photon sensitivity) [46,65].

In conclusion, SPAD and SiPM arrays can be the best candidates for pulsed-LiDAR, thanks to their fine timing resolution, high sensitivity, and relatively large number of pixels. Since assembly complexity and production costs are increasing with the introduction of 3D stacking, accurate device and electrical simulations of these detectors before fabrication is fundamental to save development time and money and to attain the expected performance. For this reason, not only TCAD-based SPAD simulators [66] and precise models of SPAD [67] and SiPM [68] have been developed, but also specific simulators for SPAD-based LiDAR systems [60].

### 2.2. Commercial SPAD and SiPM Detectors for LiDAR

In recent years, many companies (e.g., Toyota, ST-Microelectronics, Sony, Panasonic, ON Semiconductor, Ford-Argo) are developing SPAD and SiPM arrays for LiDAR applications, being deployed into more and more consumer products. 

Since 2013, Toyota (Aichi, Japan) has published work about SPAD sensors for pulsed-LiDAR, based on linear arrays of 32 macropixels, each one including 12 SPADs for photon coincidence detection [41] and also including on-chip digital signal processor (DSP) for TOF processing [42]. Toyota is also developing SPAD detectors with optimized performance for LiDAR, in particular with red-enhanced detection efficiency [43].

ST-Microelectronics (Genève, France) has developed a high-performance 3D-stacked technology, with a back-side illuminated (BSI) top tier for SPADs, connected to a high-voltage 40 nm CMOS bottom tier for front-end sensing and digital processing [44,45,46].

After having acquired SensL, ON Semiconductor (Phoenix, AZ, USA) has invested in both analog SiPM and SPAD arrays for LiDAR. The Gen3 LiDAR Imaging System is based on a 1 × 16 analog SiPM array, for sunlight outdoor application up 40 m FSR [47]; whereas “Pandion” is ON Semiconductor’s first SPAD array (with 400 × 100 pixels), suitable for large FOV LiDAR applications [48].

Panasonic (Osaka, Japan) has presented the largest SPAD array ever reported, with 1200 × 900 pixels, and a mixed dTOF—iTOF architecture for long range (250 m) LiDAR with 10 cm resolution [49]. The analog pixel is based on a vertical APD (VAPD) fabricated using BSI CMOS image sensors (CIS) technology, providing smaller pixel size (6 µm in [49]) compared to standard SPADs, thanks to vertical current flow and analog front-end. 

Recently, Sony (Tokyo, Japan) has presented a complete MEMS LiDAR system based on a 189 × 600 pixels SPAD array, in 3D-stacked technology, which includes a 90 nm BSI tier for SPADs and a 40 nm CMOS tier for digital logic [50]. The LiDAR system targets automotive applications with 300 m FSR and a micropixel logic for solar background rejection.

Princeton Lightwave (Cranbury, NJ, USA, acquired by Ford-Argo, Pittsburgh, PA, USA, in 2017), developed InGaAs/InP SPAD arrays for LiDAR at 1550 nm wavelength, with 32 × 32 pixels, cooled by thermo-electric cooling to reduce the detector noise [51].

**Figure 7:**
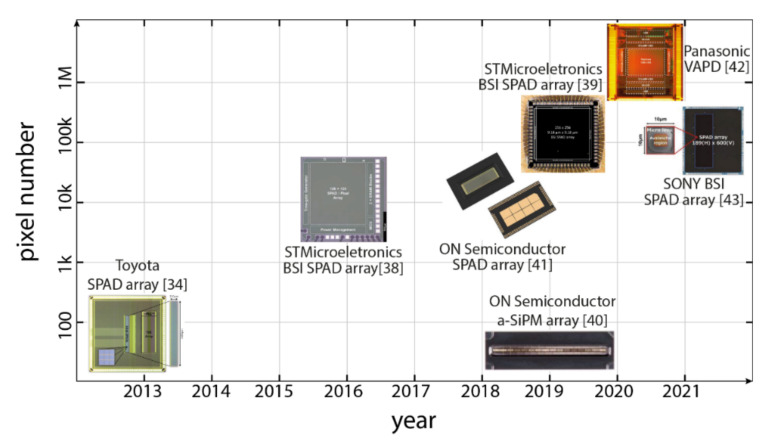
Recent trend in commercial Silicon SPAD detectors for LiDAR systems.

Figure 7 summarizes the recent trends in commercial silicon SPAD detectors for LiDAR: as can be seen, the number of pixels is constantly increasing for SPAD arrays, whereas a-SiPM arrays have a much lower pixel count. Figure 7 does not include ref. [51] since is InGaAs/InP technologies is much less mature than silicon and a fair comparison is quite difficult.

## 3. SPAD and SiPM Detectors for Pulsed-LiDAR

In this section we analyze a selection of SPAD and SiPM arrays from the literature, which present some interesting features for addressing the main challenges of pulsed-LiDAR. Table 1 lists the main characteristics of the selected chips (each detector has been named with its publication date and surname of the last author), in terms of technology node, number of pixels, number of SPAD per pixel, photon detection probability (PDP), fill-factor (FF), TDC resolution (LSB) and range (FSR). In Section 3.1 we briefly describe each architecture; then, the following sections detail how the main parameters have been optimized in the different SPAD chips.

### 3.1. Selected SPAD LiDAR Detectors Architectures

The 2013 Niclass [41] digital SiPM array, with 32 pixels of 12 SPADs each, has been one of the first specifically designed for LiDAR applications with special feature for background robustness. In fact, a fully digital coincidence detection circuit has been implemented in each pixel to signal when at least two photons are synchronously detected in a 4 ns/8 ns adjustable coincidence window. 

The 2017 Perenzoni [52] SiPM array includes 64 × 64 pixels of 8 SPADs each. The arrival-time of the first photon is timestamped by the in-pixel TDC, which can be used either with fine resolution and medium range (250 ps LSB and 6.4 µs FSR) or in altimeter mode with reduced resolution but extended range (10 ns LSB and 327 µs FSR). A coincidence circuit is implemented by counting the number of SPAD detections within a rolling time window and identifying if a certain threshold is exceeded in order to validate the TDC conversion. The chip provides both the number of photons detected within the time window (counting information) and the first photon arrival time (timing information). 

The 2018 Ximenes [53,54] detector is composed by two modules of 8 × 16 SPADs. Besides the SPADs, each module includes a selection tree, a TDC, an arithmetic logic unit (ALU) and memory bank with one word for each pixel. The tree selects the first pulse among the 8 × 16 SPADs and saves the address of the triggered SPAD. The TDC converts the first pulse’s arrival-time and the ALU combines the new TDC data with the old one, retrieved from the memory at the address corresponding to the trigger SPAD; finally, the new value overwrites the memory at the correct address. The deadtime after each event is 2.4 ns. 

The 2018 Beer [55] 192 × 2 linear array includes four SPADs per pixel, which are combined to generate an event signal when coincident detections exceeds an automatically adjustable threshold. In order to regulate the event rate, it is possible to act on the duration of the coincidence window (from 1.5 ns to 16 ns), on the coincidence threshold (from 1 to 4) and on the number of active SPADs (from 1 to 4, but higher than the coincidence threshold). Acting on these three parameters, 11 different levels of coincidences are achievable for adapting to different background levels and signal strength.

The 2019 Zhang [56] 256 × 144 SPAD array is characterized by TDC sharing and per-pixel histogramming. Each half-column (126 pixels) shares six address-latching-TDCs (ALTDCs), which are connected in daisy-chain and activated once at a time (time-stamping up to 5 photons per cycle in each half-column). A partial histogram (PH) is saved and consequently readout per each pixel. The PH consists in a reduced histogram, with only 16-bins around the distribution’s peak, which has been detected during a preliminary peak detection (PD) phase. The latter is a 3-stages phase during which only a portion of the overall TDC bits is considered (starting from the most significant bits (MSBs), down to the LSBs), and refining the peak detection in each step. At the end of this process a 16-bin histogram window is selected, and only events within that window are considered during the following acquisitions to build the PH.

The 2019 Hutchings [46] SPAD array can work either in photon counting (with full 256 × 256 resolution) or in photon timing mode, connecting groups of 4 × 4 SPADs to the same processing unit (with reduced 64 × 64 resolution). The detector is able to fast switch between the two modalities, so to perform hybrid intensity high-resolution images and 3D maps [69]. In timing mode, the detector can either work in single-hit high temporal resolution mode (38 ps resolution) or in multi-event histogramming mode (560 ps resolution). In the latter, a histogram records events that exceed an adjustable threshold of coincident detections among the 16 SPADs belonging to the same group.

In a 2020 Seo paper [57] a 63 channel SiPM array (with four SPADs per pixel) is used in a line scanning setup. Each pixel includes two histogramming TDCs (hTDCs): a 5-bit coarse TDC with a shift register architecture used to build the analog coarse histogram through a current injection in the corresponding accumulator and a 6-bit fine TDC, based on delay-locked loop (DLL), which is used to feed a fine histogram. Both hTDCs are multi-hit (i.e., can be triggered multiple times during the same laser period) to achieve high throughput. The LiDAR system is based on two laser diodes, which emit two successive pulses, with a delay which represents the ID identifier of the system for multicamera operation. When coincident events among the SPADs in the same pixel are detected, counts are accumulated in the coarse histogram, which is consequently digitalized by comparing the voltages accumulated into each bin with a reference voltage. The dedicated logic for interference filtering, detects the two peaks with a delay corresponding to the device ID and selects the time widow during which the fine TDC is enabled to build the fine histogram. Combining the information of coarse and fine histogram, long range and high precision are achieved.

The 2021 Padmanabhan [58] chip is composed by 256 × 128 pixels, grouped in 16 × 16 SPADs clusters, but preserving the native full imaging resolution (256 × 128). Each pixel cluster have one TDC, which timestamps the first photon, whereas the conversion is validated only if other pixels within the cluster are triggered within an adjustable (from 500 ps to 2.2 ns) coincidence window. The number of coincidences to validate measurements is also adjustable over seven levels, and the number of detected photons within the coincidence window is stored in a memory and readout along with TDC conversion and first triggered pixel address. Progressive gating can be enabled for further time filtering the incoming signal, when the target depth is roughly known.

In the 2021 Kumagai [37] chip, 183 × 600 SPADs can be combined in macropixels with either 3 × 3 or 4 × 4 SPADs each. A coincidence detection circuit performs background suppression, the arrival-times converted by the TDC accumulate in the TOF histogram, and finally a finite impulse response (FIR) filter detects the peak of the histogram.

### 3.2. Maximum Range and Precision

In the presented architectures, the maximum range is mainly defined by the TDC FSR, and the measurement precision by the TDC resolution (i.e., LSB) and the laser pulse-width. In order to avoid distance ambiguity for object farther than the maximum range, either the TDC should not refold when its FSR is exceeded, or photon detections must be enabled within a time window shorter than the TDC FSR by means of detector gating.

Note that, with a priori rough knowledge of the absolute target distance, the TDC FSR limits only the image depth range and not the maximum target distance, in fact the laser trigger (typically corresponding to the TDC START signal) can be properly delayed in order to fit the target distance profile within the TDC range [32].

In many pulsed-LiDAR systems, the centroid of the arrival-times histogram is computed to estimate the TOF, in order to achieve a precision not limited by the TDC quantization error. In fact, the precision improves as √N, where *N* are the counts within the histogram peak. In order to reduce data-throughput, the histogram can be computed on-chip, as in Hutchings [46], typically with the limitation of having a reduced FSR (in [46] the FSR reduces to 9 ns with on-chip histogram computation). Partial histograms (PHs) around the peak, detected through a coarse histogram, can be computed as in Zhang [56] and Seo [57] in order to preserve both high resolution and long range.

Precision and maximum range can be also traded-off depending on the application. For instance, in Perenzoni’s work [52] TDC can be operated in two modes: one achieves high resolution and short range by using a coarse counter combined with a fine counter, the other attain low resolution and long range by cascading the two counters in a unique coarse counter with longer bit-depth.

### 3.3. FOV and Angular Resolution

FOV and angular resolution in staring setups depend on the number of pixels. Modern 3D stacking has allowed to fabricate SPAD arrays with definitely higher pixel number in respect to planar technologies, reaching hundreds of kilo-pixels in the same chip. In Hutchings’ study [46], the image resolution has been improved by combining 2D high resolution imaging (each of the 256 × 256 SPADs works independently) with 3D lower resolution maps (SPADs are combined in macropixels, with 4 × 4 detectors each). Since 2D and 3D information cannot be simultaneously acquired (because some hardware resources are shared by the two modalities), the detector is used in a hybrid mode with interleaved 2D and 3D frames, as shown in [69]. Data-fusion proposed in [70], further allows to improve image resolution, by combining information from a time-resolved 240 × 320 pixels SPAD array with a co-registered high-resolution red-green-blue (RGB) digital camera. Assuming that close regions have local correlations in depth and exploiting color and depth information, high resolution depth information is gathered from a small subset of data. 

FOV and angular resolution can be further increased using scanning techniques. For instance, in Niclass [41] the 32-pixels linear array has been used in combination with a 3-facet polygonal rotating mirror to achieve a final resolution of 340 × 96 pixels corresponding to 170° × 4.5° FOV. In Ximenes [53,54], a 256 × 256 image resolution is obtained through a dual axis laser scanner using an 8 × 32 SPAD array. In Seo [57], a 1 × 36 SiPM is used for line scanning through MEMS and rotating polygon mirrors to achieve 2200 × 36 resolution and 120° × 8° FOV. In Kumagai [50], a blade illumination laser scanning with horizontally oscillating MEMS mirrors is used in combination with a staring 63 × 200 SPAD camera activated one column at a time, in a non-coaxial setup.

Since the desired FOV and angular resolution strongly depend on the application, sometimes data from high resolution cameras are combined in post processing, in order to achieve higher signal (i.e., better centroid precision or longer range) and narrower angular resolution. An example is shown in the Perenzoni [52] detector designed for spacecraft navigation and landing. In fact, the application requires both 3D imaging with a spatially resolved camera, but also single point altimetry performed with a laser collimated in a single spot. Thus, a 64 × 64 imager has been designed for 3D imaging, but pixels information can also be combined for single-point long-range altimetry.

### 3.4. Measurement Speed and Background Suppression

Measurement speed is a fundamental requirement for real-time applications. Flash-LiDAR architectures, not needing slow scanning, represent the best candidates for such exploitations. Nevertheless, high acquisition speed requires also high photon detection efficiency (PDE=PDP�FF) to detect as much signal as possible (note that in flash LiDAR with flood illumination the energy per pixel is typically very weak), low detector dead-time to achieve high counting rates and finally high TDC conversion rate not to waste photons. Typically, the bottleneck is due to the TDC, which is able to timestamp only the first photon in most SPAD arrays. Saturating either detector or TDC leads to photon “pile-up” distortion [71], i.e., it becomes impossible to reconstruct the real returning signal shape through TCSPC, because the first arrived photons mask the following ones. This problem is exasperated by the presence of strong (e.g., solar) background illumination. In fact, background photons alone can be so many to saturate the detector (or the TDC) even before the detection of the laser return photons, especially when close to FSR end.

A typical method used to remove the effect of background is to compute the arrival-time histogram. Being the background equally distributed in time, it generates a flat noise floor. Therefore, by considering only the dominant peak (due to the laser pulse) for the centroid computation, the background is automatically removed. When accumulating the histogram, the timestamps measured by the TDCs are used as the addresses of the bins in the histogram storage memory, while the content of a memory address (histogram depth) represents how many times that address has been hit. Per-pixel complete histogram computation becomes unfeasible due to chip area constraints of large SPAD arrays. For instance, for a 15-bit TDC, 10-bit histogram depth, and 128 × 128 pixels, the required memory would be about 2^15^ · 10 · 128^2^ = 5.4 Gb, corresponding to about 1 cm × 1 cm chip area in a 90 nm technology. Thus, solutions based on partial histograms (PHs) have been proposed. In Zhang [56] the proposal is to build a PH by limiting the histogram window only to 16 bins around the distribution peak, which has been detected during the preliminary PD phase. All timestamps outside the histogram peak are discarded from the in-chip PH, but they can be readout separately, in order to reconstruct the full histogram in post processing. In Seo [57], a PH is obtained through the combination of 5-bit coarse histogram for peak detection and a 6-bit fine histogram for fine resolution. A similar solution, implemented in a field-programmable gate-Array (FPGA), is presented in [72]. In that case, two separate histograms are computed, dividing the timestamp value in two parts: the coarse histogram (CH) considers only the MSBs of all the timestamps provided by the TDC, and it is used to find the peak of the distribution; whereas the fine histogram (FH) considers just the LSBs of timestamps centered around the peak, and it is used to achieve fine depth resolution.

Nevertheless, as already mentioned, when saturation occurs, pile-up distortion prevents to accumulate a correct histogram. For instance, Zhang’s device [56] works only with very low photon rates, because the address of the triggered SPAD can be correctly detected only if none of the other pixels in the same half-column gets triggered at the same time. In fact, a collision detection bus is implemented to discard events related to coincident detections, which would provide a wrong address. In fact, instead of using binary addresses, each address is constituted by four ones and four zeros; in case of collisions, the resulting address would have more than four zeros so it can be easily discarded. An increased incoming photon rate would increase also the collision probability in half column, thus reducing the effective count rate and causing histogram distortion. 

For this reason, most of the SPAD and SiPM arrays designed for LiDAR introduce some special features based on photon coincidence to reduce the impact of background and photon pile-up. The general idea consists of triggering the TDC only when multiple photons are detected within a short coincidence time window, because laser photons are confined within the pulse width whereas background photons are usually equally distributed in time. For instance, in Niclass [41], Seo [57] and Kumagai [50], the macropixel triggers the TDC only when a coincidence event is detected, whereas in *2021 Padmanabhan* [48] the TDC is triggered by the first detected photons but the conversion is validated only if a given number of coincident photons are detected within an adjustable (500 ps–2.2 ns) coincidence window. In Perenzoni [52], the SPAD output pulse is shrunk down to about 260 ps and all shrunk pulses of the same pixel are combined through an OR-tree into a single pulse train. Finally, such pulses feed a digital triggering logic, which identifies if the number of incoming photons exceeds a given threshold within a rolling time window, and in that case the TDC gets triggered. A similar approach is used also in Hutchings [46], but there also the conversion rate is increased by using a multi-event TDC (i.e., able to detect more than one event per frame) and an in-pixel histogrammer. Thus, Hutchings [46] represents a huge step forward in high-speed real-time LiDAR, being a multipixel camera suitable for flash-LiDAR and being optimized for very high counting and conversion rates. Another architecture where the TDC is not limited to convert only the first photon is Ximenes [53,54]: in fact, an ALU combines multiple timestamps coming from the same pixel. However, in that architecture, the TDC is shared among 128 pixels and only the first event among them is converted with a 2.4 ns dead-time between two subsequent conversions. Furthermore, that architecture does not apply any technique to prevent background events to trigger the TDC, thus pile-up distortion may impair array functionality with high solar illumination. 

A limitation of all aforementioned architectures is that the coincidence threshold is fixed a priori and is very critical, since too low a value leads to a non-optimal background rejection, whereas too high a value drastically reduces the event detection, loosing also signal events. In Beer [52], an automatically adjusting coincidence level is proposed to dynamically adapt the detector dynamic range to the illumination condition of the scene. Background is measured during the time-windows when the laser is off, between two successive pulses, through an 8-bit counter; by regulating the coincidence window, the number of active SPAD per pixel, and the coincidence threshold, the detector event rate can be kept within a desired range, fixed by the user. The main limitation of that architecture is the slowness in adapting to considerable changes in illumination levels, since the system takes about 0.4 s to adjust the coincidence settings from lowest to highest level.

In Padmanabhan [58], temporal gating is used in combination with TCSPC for time filtering the incoming light, when the target distance depth is roughly known. As proposed in [71], gating is exploited to introduce different temporal offsets between laser cycles and SPAD acquisition windows, thus enabling photons detections in later time bins that would otherwise have been masked by early arriving ambient photons. This distributes the effect of pile-up across all histogram bins, thus eliminating the first-photon distortions. The two best approaches, defined in [71], consist in uniform deterministic shifts of the SPAD activation window with respect to laser emission, and in photon-driven shifts implemented by making the SPAD dead-time longer than the laser period and relying on the mismatch to automatically cycle through all possible shifts. In addition, [71] proposes optimization algorithms to recover the incident waveform from the distorted histogram, gaining an overall improvement up to 30% in the relative root mean square error.

Other optical methods for background suppression (independent of the detector) can be exploited as reported in [73]. Those methods include narrow-spectrum light sources with spectral components where the background light has low power density, coupled with narrow optical bandpass filters in order to optically block the incoming background light. However, due to laser technology limitations, typically 5 nm to 40 nm bandpass filters are used, not yet able to definitely suppress background. Another optical method confines the emitted optical signal to a small area (or multiple areas withing the FOV) and then correspondingly images that area onto the sensor. This can be achieved for instance by means of diffractive optical elements (DOEs), which generate spot illuminations of the scene. As demonstrated in [74], scanning can be convenient in terms of signal-to-background ratio (SBR) in respect to flash-LiDAR. In fact, given a certain amount of energy per pulse and total measurement time, scanning across M positions reduces the exposure time by a factor M, but it is compensated by the fact that the laser energy is concentrated in smaller area thus the energy per area density increases by a factor M as well. Hence, in the scanning setup, the signal has the same intensity as in flash-LiDAR, the background is collected by a smaller area, thus improving SBR. 

### 3.5. Eye-Safety

Eye-safety regulation provides a limit to the maximum laser energy, strongly dependent on laser wavelength. Wavelengths longer than 1400 nm are preferred, because they are absorbed by the anterior segment of the eye thus preserving the retina. Nevertheless, 905 nm is commonly used in LiDAR application, mainly due to pulsed laser and detectors availability and because of the lower absorption by water (thus allowing LiDAR measurements even through fog and rain) compared to 1550 nm, which is the other standard wavelength employed in LiDAR. In fact, nowadays great efforts are focused on developing Silicon detectors with enhanced sensitivity at 905 nm [43,75] and high FF through 3D stacking (e.g., Ximenes [53,54] and Hutchings [46]) and on designing eye-safe certified LiDAR systems, such as Kumagai [50]. 

### 3.6. Interference Robustness for Multi-Camera Operation

Multicamera LiDAR operation is a requirement typically dealt with at system level, and does not strictly depend on the detector. Possible solutions are described in [73], consisting in space-division (SDMA), wavelength-division (WDMA), time-division (TDMA), frequency-division (FDMA), and code-division (CDMA) multiple access. In SDMA, each LiDAR detector has a different FOV, so different systems cannot interact; hence, it is applicable only in very well controlled environments (e.g., in industrial automation). WDMA consists of using different illumination wavelengths and narrow band-pass filters on each detector; it can be applied on a limited number of concurrently working systems which use different wavelengths. TDMA relies on synchronized cameras working in different time slots. In FDMA, applicable only to CW-LiDAR, each system has its own modulation frequency and, as for WDMA, the number of interfering cameras is limited. In CDMA different temporal signatures are used for the illumination, then the returning signal is demodulated accordingly to the known source pattern.

An example of CDMA implementation is presented by Ximenes [53,54], which exploits a laser signature identification technique, based on a digital polar modulation based on Phase-Shift Keying (PSK) on the outgoing light and on the detected photon timing information for proper signal recovery. As a result, any light source other than its own, is spread flat across the arrival-times histogram. A simplified approach is presented in Seo [57], where a dual laser source emits two pulses delayed by a precise time interval, which represents the identifier of the LiDAR system; then, only peak couples with the proper time delay are considered valid for the TOF estimation. This simplified architecture can also be implemented in a reduced number of interfering systems (e.g., 32 in [57]). 

## 4. Discussion on Next Generation Detectors

SPAD arrays are emerging as the best candidates for long-range high-speed pulsed-LiDAR, thanks to their precise timing and single photon sensitivity. A classical limitation of SPAD arrays compared to other technologies (e.g., EM-CCDs and I-CCDs) is the reduced number of pixel (hence reduced angular resolution from a given FOV). However, such a limitation is partially overcome thanks to BSI and 3D stacking technologies, which allow to shrink pixel pitch and increase pixels number, reaching up the Mpixel level.

Fine timing resolution (typically in the order of few tens of picoseconds) and low time-jitter detectors and electronics allow to reach centimeter precision, which can be further improved (down to millimeters) by collecting repetitive measurements and computing the histogram centroid. 

Although single photon sensitivity, typical of SPAD detectors, is a welcome feature in LiDAR (especially for long ranges and low reflectivity targets), saturation due to high background limits or completely prevents TOF measurements. For these reasons, high DR is a key requirement for LiDAR detectors. In this paper, we discussed and compared many different architectures, mainly based on SiPM arrays or cluster of spatially resolved SPADs, which implement background suppression for enhancing the detector DR.

Eye-safety and multicamera operation are critical requirements for outdoor LiDAR systems and are typically managed at system level, through optimized illumination wavelength and temporal patterns.

Table 2 lists the performance of the selected SPAD arrays when used in LiDAR applications. Medium ranges (about 50 m FSR) and centimeter precisions can be reached, at least theoretically, by most of those architectures. However, the actual achievable range and precision depend not only on the detection chip but also on laser power, target reflectivity, and background. In respect to flash-LiDAR, scanning setups typically provide higher image resolution (few kilopixels in flash LiDAR vs. tens of kilopixels in scanning setups), thus better angular resolution for a given FOV, at the expense of lower frame rate (about 30 fps in flash-LiDAR vs. typically less than 10 fps in scanning setups).

Next generation LiDAR detector should take advantage of BSI and 3D stacking in order to design optimized SPADs at the top tier (with enhanced PDP at 900 nm and almost 100% FF through microlenses) and advanced processing electronics in a scaled technology at the bottom tier. Detectors with both high image resolution photon counting (in which each SPAD works independently) and low-resolution photon histogramming (in which SPADs are clustered for background rejection) are suitable for enhancing image quality through data-fusion and image processing.

Most of the presented architectures reject background through on-chip coincidence detection. However, the coincidence threshold is the same for the entire array and is fixed during the entire measurement. A definitive improvement would be to implement an automatic-adjustable threshold at the pixel-level, by checking counting information, so to keep the coincident event rate within the best operative zone (with no signal events loss, while filtering out background). Partial histograms are typically used for resource optimization, but they are not that effective in preventing pile-up distortion; whereas detector progressive gating performs time filtering of incoming light, preventing detector saturation, but increases the measurement time since the gate window must shift across the entire FSR. Combination of partial-histogram (for peak detection) and detector gating centered around the histogram peak would contribute to avoid background-induced saturation without increasing the measurement time.

Taking inspiration from quantum imaging, in which quantum states are used to improve image resolution and sensitivity, a similar principle can be implemented in future LiDAR detectors able to detect photon coincidence and to discriminate classical background illumination light from source’s quantum states [76].

## 5. Conclusions

We have analyzed different LiDAR techniques, illumination schemes and detectors technologies and we found that the best trade-off for long-range high-speed LiDAR is employing SPAD arrays in pulsed-LiDAR systems. Both scanning- and flash-LiDAR systems have pros and cons. Scanning provides better SBR, since the background collection area is reduced through smaller detectors (typically linear arrays), whilst flash-LiDAR attain higher framerates. We focused on nine selected SPAD array architectures, to show how they face the main LiDAR challenges. Particularly high solar background and, consequently, pile-up distortion may prevent to compute the actual TOF. Histogramming and coincidence detection, even employed in combination, are the most common techniques used to make detectors robust to background.

Although in this paper we focused on SPAD detectors for LiDAR applications, same or similar architectures can be employed also for many other applications exploiting TCSPC to reconstruct very fast and faint optical waveforms, such as FLIM and spectroscopy [77,78].

## Figures and Tables

**Table 1:** Main specifications of selected SPAD and SiPM arrays for pulsed-LiDAR.

Detector	Technology (nm)	Pixel Number	SPADs per Pixel	PDP @ 905 nm (%)	FF (%)	TDC LSB (ps)	TDC FSR (ns)
2013 Niclass [41]	180	32 × 1	12	N.A.^1^	70	208	853
2017 Perenzoni [52]	150	64 × 64	8	N.A.^1^	26.5	250 10,000	6400 327,000
2018 Ximenes [53,54]	BSI 45/65	8 × 32	1	7.5	31	60	1000
2018 Beer [55]	350	192 × 2	4	2	5.3	312.5	N.A.
2019 Zhang [56]	180	252 × 144	1	5	28	48.8	200
2019 Hutchings [46]	BSI 90/40	256 × 256 ^2^ 64 × 64 ^3^	1 ^2^ 4 × 4 ^3^	5	51	38 ^4^ 560 ^5^	143 ^4^ 9 ^5^
2020 Seo [57]	110	1 × 36	4	N.A.^1^	N.A.^1^	156	320
2021 Padmanabhan [58]	BSI 45/N.A. ^1^	256 × 128	1	N.A.^1^	N.A.^1^	60	1000
2021 Kumagai [50]	BSI 90/40	63 × 200 31 × 100	3 × 3 4 × 4	22	N.A.^1^	1000	1800

^1^ Not Available. ^2^ Only photon counting. ^3^ Photon timing. ^4^ Single-hit mode. ^5^ Histogramming.

**Table 2:** Selected SPAD and SiPM arrays performance in LiDAR applications.

Detector	Maximum Range (m)	Precision (cm)	FOV	Angular Resolution	Image Resolution	Frame Rate (fps)	Optical System
2013 Niclass [41]	128	3.8	170° × 4.5°	0.5° × 0.05°	340 × 96	10	2D Scanning
2017 Perenzoni [52]	367 ^2^ 5862 ^3^	20 ^2^ 50 ^3^	N.A.^1^	N.A. ^1^	64 × 64	7.7 ^2^ 7.2 ^3^	Flash
2018 Ximenes [53,54]	7 80	15 47	7° × 7°	0.02° × 0.02°	256 × 256	0.031	2D Scanning
2018 Beer [55]	6.5 ^4^	4.7	36° × 1°	0.2° × 1°	36 × 1	25	Flash ^5^
2019 Zhang [56]	50	0.14	40° × 20°	0.15° × 0.14°	252 × 144	30	Flash
2019 Hutchings [46]	50	N.A.^1^	1.2° × 1.2°	0.02° × 0.02°	64 × 64	30	Flash
2020 Seo [57]	48	0.85	120° × 8°	0.05° × 0.2°	2200 × 36	1.18	1D Scanning
2021 Padmanabhan [58]	10 ^6^	N.A.	2° × 2°	0.16° × 0.16°	128 × 128	N.A.	Flash
2021 Kumagai [50]	150 300	15 30	25.2° × 9.45°	0.15° × 0.15°	168 × 63	20	1D Scanning ^7^

^1 ^Not Available. ^2^ Imaging mode. ^3^ Altimeter mode. ^4^ Target distance in the measure reported in the paper (maximum range is N.A.). ^5^ Can be used also for 1D scanning. ^6^ Theoretical FSR 100 m ^7^ Staring camera, one column at a time is activated.

## Data Availability

Not applicable.
